# L-Carnitine Suppresses Transient Receptor Potential Vanilloid Type 1 Activation in Human Corneal Epithelial Cells

**DOI:** 10.3390/ijms241411815

**Published:** 2023-07-23

**Authors:** Alexander Lucius, Sirjan Chhatwal, Monika Valtink, Peter S. Reinach, Aruna Li, Uwe Pleyer, Stefan Mergler

**Affiliations:** 1 Klinik für Augenheilkunde, Charité—Universitätsmedizin Berlin, Corporate Member of Freie Universität Berlin and Humboldt-Universität zu Berlin, 13353 Berlin, Germanyaruna.li@charite.de (A.L.);; 2 Faculty of Medicine, Institute of Anatomy, TU Dresden, 01216 Dresden, Germany; 3 Equality and Diversity Unit, Faculty of Medicine, TU Dresden, 01307 Dresden, Germany; 4 School of Ophthalmology and Optometry, Wenzhou Medical University, Wenzhou 325027, China

**Keywords:** human corneal epithelium, transient receptor potential channel vanilloid 1, L-carnitine, intracellular Ca^2+^ signaling, planar patch-clamp technique, cell volume, hypertonic cell shrinkage

## Abstract

Tear film hyperosmolarity induces dry eye syndrome (DES) through transient receptor potential vanilloid type 1 (TRPV1) activation. L-carnitine is a viable therapeutic agent since it protects against this hypertonicity-induced response. Here, we investigated whether L-carnitine inhibits TRPV1 activation by blocking heat- or capsaicin-induced increases in Ca^2+^ influx or hyperosmotic stress-induced cell volume shrinkage in a human corneal epithelial cell line (HCE-T). Single-cell fluorescence imaging of calcein/AM-loaded cells or fura-2/AM-labeled cells was used to evaluate cell volume changes and intracellular calcium levels, respectively. Planar patch-clamp technique was used to measure whole-cell currents. TRPV1 activation via either capsaicin (20 µmol/L), hyperosmolarity (≈450 mosmol/L) or an increase in ambient bath temperature to 43 °C induced intracellular calcium transients and augmented whole-cell currents, whereas hypertonicity induced cell volume shrinkage. In contrast, either capsazepine (10 µmol/L) or L-carnitine (1–3 mmol/L) reduced all these responses. Taken together, L-carnitine and capsazepine suppress hypertonicity-induced TRPV1 activation by blocking cell volume shrinkage.

## 1. Introduction

Dry eye syndrome (DES) is a common ocular disorder whose prevalence can be as high as 75 % amongst adults over 40 years of age, with women more frequently affected than men [1,2,3]. This condition increasingly affects more younger individuals than older individuals since younger people frequently tend to spend more time viewing displays on mobile devices and computer screens; this can reduce blinking frequency, with consequential increases in tear film osmolarity [4]. Different symptomatic treatments are available for topical application, e.g., multiple-action tear substitutes [5], anti-inflammatory drugs such as cyclosporine A [6], or eye drops containing osmoprotectants [7] like L-carnitine [8,9,10]. Despite the described efficacy of carnitine supplementation in the treatment of DES, it is unknown whether an interaction with the transient receptor potential vanilloid 1 channel (TRPV1) underlies its modes of action in the corneal epithelium.

A hyperosmolar tear film can trigger the inflammatory cycle of DES [11,12,13]. Notably, a hyperosmolar microenvironment also activates the osmosensitive TRPV1 channel, which is expressed in different ocular tissues [14,15,16]. In addition to hyperosmolarity [17,18], temperatures above 43 °C (“heat sensor”) [19], declines in pH (“pain sensor”) [20], or exogenous pharmacological stimuli such as capsaicin (CAP, “capsaicin receptor”) [21] can also induce transient increases in intracellular calcium levels by activating TRPV1 on the cell membrane. Furthermore, the activation of TRPV1 in epithelial and conjunctival cells on the ocular surface by a hyperosmolar tear film elicits a signaling cascade that induces increases in proinflammatory cytokine expression and leads to the release of interleukins (IL)s 6 and 8 [22,23,24], thereby contributing to the inflammatory cycle of DES. TRPV1 activation also promotes ocular scarring and tissue fibrosis, which can impair visual acuity [25]. Therefore, efforts are underway to identify agents that inhibit DES symptomology through the selective inhibition of TRPV1 activation.

The liver synthesizes L-carnitine, a substance which is indispensable in the metabolism of fatty acids. It also has osmoprotective properties and can reduce apoptosis rates in response to hypertonicity in the human corneal epithelium [26]. The uptake of L-carnitine into the cells of the human corneal epithelium is regulated by the organic cation transporter 2 (OCTN2) in a time-dependent manner, requiring Na^+^ and a moderately acidic pH [26]. These results account for efforts to identify the mechanisms of action of osmoprotectants like L-carnitine (i.e., Carnidrop, OPTIVE^TM^, and OPTIVE Plus^®^) [9,27,28,29,30,31,32] that can improve the therapeutic management of DES [29,33].

Previously, we showed that L-carnitine acting as an osmoprotectant suppressed hypertonicity-induced TRPV1 activation in human conjunctival epithelial cells (HCjEC) and human corneal keratocytes (HCK) [34,35]. In the current study, we also show that L-carnitine inhibits another biomarker of TRPV1 activation; namely, it also inhibits these rises through blockage of TRPV1 activation. This correspondence between the inhibitory effects of L-carnitine and capsazepine (CPZ) confirms that they both block hypertonic-induced TRPV1 activation. Such correspondence accounts for the efficacy and safety of L-carnitine’s use as an eyedrop supplement for treating DES.

## 2. Results

### 2.1. Functional Validation of TRPV1 Expression

**Figure 1:**
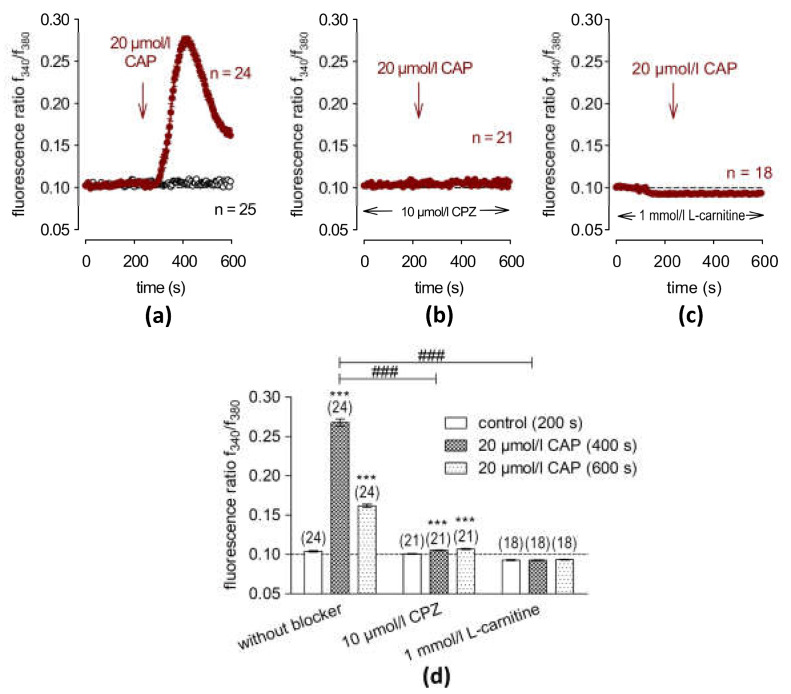
L-carnitine (1 mmol/L) suppresses CAP-induced Ca^2+^ increases. The time-dependent changes are shown as relative intracellular Ca^2+^ levels in fura2-loaded HCE-T cells. Data are represented as means ± SEMs, and n indicates the number of cells examined in this set of experiments. The dashed line represents the reference line for the baseline value (0.1). Arrows indicate the extracellular application of CAP at 240 s. (**a**) An amount of 20 μmol/L of CAP induces increases in Ca^2+^ entry (n = 24; filled circles). As control without CAP application, no changes in intracellular Ca^2+^ level could be observed (n = 25) (open circles). (**b**) Representative graph of the TRPV1 blocking effect of 10 μmol/L of CPZ on CAP-induced Ca^2+^ influx (negative control) (n = 21) (**c**) 1 mmol/L of L-carnitine completely blocked the CAP-induced Ca^2+^ increase (n = 18). (**d**) Statistical analyses of the CAP- induced Ca^2+^ response patterns with and without CPZ (10 µmol/L) or L-carnitine (1 mmol/L). Bars represent mean values ± SEMs of the fluorescence ratio at 200 (control), 400 and 600 s (CAP). The numbers of cells measured in both experiments are indicated in brackets above the bars. The asterisks (*) indicate statistically significant differences with and without CAP (n = 18 to n = 24; *** *p* < 0.001; paired tested). The hashtags (#) refer to unpaired data with and without CPZ or L-carnitine (^###^ *p* < 0.001).

SV40-immortalized HCE-T cells are a relevant *in situ* model for the human corneal epithelium because capsaicin (CAP) induces Ca^2+^ transients that are like those induced in primary corneal epithelial cell cultures [23]. In the current study, 20 µmol/L of CAP induced an increase in the f340 nm/f380 nm fluorescence ratio from 0.1040 ± 0.0009 at t = 200 s to 0.2677 ± 0.0044 at t = 400 s (n = 24, *** *p* < 0.001), which partially recovered at the end of the experiment (0.1617 ± 0.0022 at t = 600 s; n = 24, *** *p* < 0.001) (Figure 1a). A similar result was also observed in previous experiments which used a different calcium imaging setup (Figure S1a). In addition, planar patch clamping confirmed CAP-induced increases in whole-cell currents (20 µmol/L of CAP) (Figure S2). As a negative control, 10 µmol/L of CPZ [36] was used as a corresponding TRPV1 blocker, which clearly suppressed the CAP-induced Ca^2+^ increase after 400 s to 0.1050 ± 0.0007, t = 400 s, (n = 21, ^###^ *p* < 0.001) (Figure 1b). Taken together, these results confirm that TRPV1 channels are functionally expressed in HCE-T cells.

### 2.2. L-carnitine Suppresses CAP-Induced Increases in Ca^2+^ and Whole-Cell Currents

The effect of 1 mmol/L of L-carnitine was determined after a 30 min pre-incubation in the isotonic RLS medium on the CAP-induced increases in the f340 nm/f380 nm ratio. While CAP alone induced a transient increase (Figure 1a), 1 mmol/L of L-carnitine blocked this increase since the f340 nm/f380 nm ratio remained close to the baseline (f340/f380 = 0.0934 ± 0.0005; t = 600 s; n = 18, ^###^ *p* < 0.001) (Figure 1c). Three mmol/L of L-carnitine had the same inhibitory effect (f340/f380 = 0.1334 ± 0.0082; t = 600 s; n = 48, ^###^ *p* < 0.001). The same result was obtained from another calcium imaging setup mentioned in the supplement (Figure S3a,b). Statistical analyses in Figure 1d and Figures S1c and S3b,d,f show that 1–3 mmol/L of L-carnitine suppressed the CAP-induced Ca^2+^ increase. In addition, 3 mmol/L of L-carnitine also suppressed CAP-induced increases in the whole-cell current measurements (Figure S2).

### 2.3. Hypertonic Challenge Activates TRPV1

**Figure 2:**
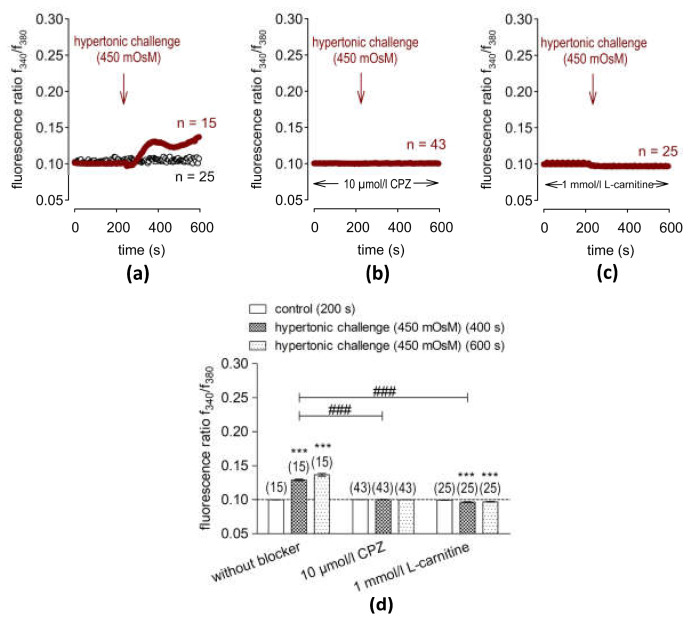
Suppression of hypertonicity-induced increases in intracellular Ca^2+^ levels by CPZ (10 µmol/L) and L-carnitine (1 mmol/L). The time-dependent changes are shown as relative intracellular Ca^2+^ levels in fura2-loaded HCE-T cells. Data are represented as means ± SEMs, and n indicates the number of cells examined in this set of experiments. The dashed line represents the reference line for the baseline value (0.1). Arrows indicate the extracellular application of the hypertonic solution (RLS) at 240 s. (**a**) A hypertonic challenge (450 mosmol/L) induced an increase in Ca^2+^ influx in HCE-T cells (n = 15; filled squares), whereas non-treated control cells maintained at the baseline level (n = 25; open circles). (**b**) The same experiment as shown in (**a**), but in the presence of 10 μmol/L of CPZ, showing a complete blocking of hypertonic challenge-induced Ca^2+^ increase (negative control) (n = 43). (**c**) An amount of 1 mmol/L of L-carnitine abolished the hypertonicity-induced Ca^2+^ increase (n = 25). (**d**) Statistical analyses of the hypertonicity-induced Ca^2+^ response patterns with and without CPZ (10 µmol/L) or L-carnitine (1 mmol/L). Bars represent mean values ± SEMs of the fluorescence ratio at 200 (control), 400 and 600 s (hypertonicity). The numbers of cells measured in both experiments are indicated in brackets above the bars. The asterisks (*) indicate statistically significant differences with and without CPZ or L-carnitine (n = 15–43; *** *p* < 0.05 at the minimum; paired tested). The hashtags (#) refer to unpaired data with and without CPZ or L-carnitine (^###^ *p* < 0.001).

Next, the effects of exposure to the hypertonic (≈450 mmol/L) medium on intracellular Ca^2+^ levels, which are used as additional biomarkers of TRPV1 functionality [17,35], were assessed. Similar to the increase induced by 20 µmol/L of CAP, the baseline f340 nm/f380 nm fluorescence ratio rose from 0.0999 ± 0.0002 at t = 200 s (n = 15) to 0.1291 ± 0.0013 at t = 400 s (n = 15, *** *p* < 0.001) and reached 0.1368 ± 0.0019 at the end of the experiment (t = 600 s, n = 15, *** *p* < 0.001) (Figure 2a). Pre-incubation with CPZ (10 µmol/L) completely suppressed the hypertonicity-induced increase in the Ca^2+^ influx (0.1003 ± 0.0002 at t = 400 s, n = 43, ^###^ *p* < 0.001) (Figure 2b). Overall, these results validate that exposure of HCE-T to hypertonic stress induces a described response that is indicative of functional TRPV1 expression in HCE-T cells [17,18].

### 2.4. L-Carnitine Suppressed Hypertonicity-Induced Increases in Intracellular Ca^2+^

Like CPZ, 1 mmol/L of L-carnitine suppressed the 450 mmol/L hypertonic challenge increase in the f340 nm/f380 nm fluorescence ratio to 0.0968 ± 0.0005 at t = 600 s (n = 25, ^###^ *p* < 0.001) (Figure 2c, d). Three mmol/L of L-carnitine had a similar inhibitory effect (0.1028 ± 0.0028 at t = 600 s; n = 30, ^###^ *p* < 0.001) (Figure S3d).

### 2.5. L-Carnitine Suppresses Heat-Induced Ca^2+^ Transients

**Figure 3:**
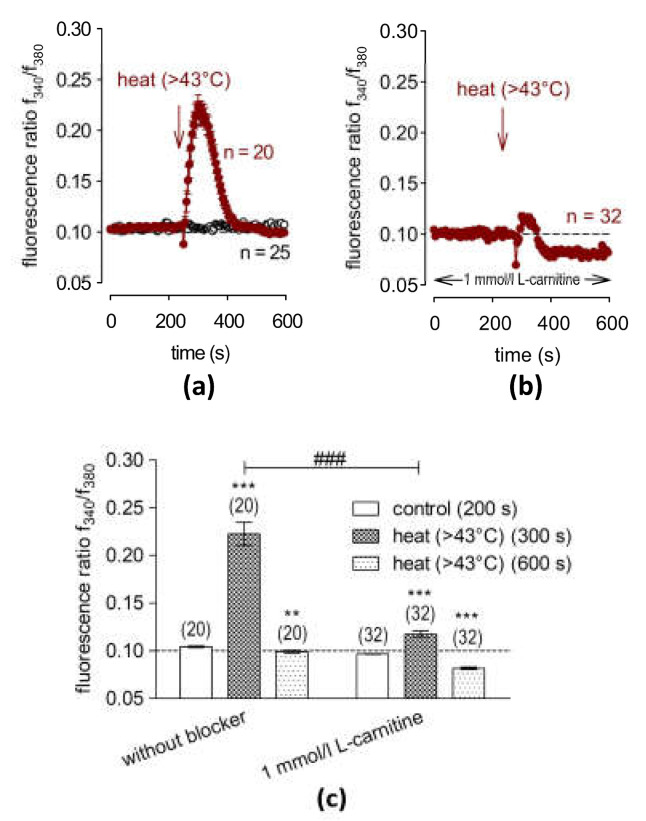
L-carnitine (1 mmol/L) suppresses heat-induced increases in [Ca^2+^]_i_ levels. The time-dependent changes are shown as relative intracellular Ca^2+^ levels in fura2-loaded HCE-T cells. Data are represented as means ± SEMs, and n indicates the number of cells examined in this set of experiments. The dashed line represents the reference line for the baseline value (0.1). Arrows indicate the application of the heated solution (RLS) at 240 s. (**a**) A heat pulse (1–2 min duration) (>43 °C) induced an increase in Ca ^2+^ influx in HCE-T cells (n = 20; filled squares), whereas non-treated control cells showed constant baseline measures (n = 25; open circles). (**b**) The same experiment as (**a**) but in the presence of 1 mmol/L of L-carnitine, which clearly suppressed the heat-induced increases in the Ca^2+^ level (n = 32). (**c**) A summary of the statistical analysis of the experiments with heat and 1 mmol/L of L-carnitine. Bars represent mean values ± SEMs of the fluorescence ratio at 200, 300 and 600 s. The numbers of cells measured in both experiments are indicated in brackets above the bars. The asterisks (*) designate significant increases in [Ca^2+^]_i_ with heat stimulation (t = 300 s; n = 20–32; ** *p* < 0.01 at the minimum; paired tested) compared to the control (t = 200 s). The hashtags (#) indicate statistically significant differences in fluorescence ratios between heat application and 1 mmol/L of L-carnitine (t = 300 s and 600 s; n = 20–32; ^###^ *p* < 0.001; unpaired tested).

An elevation of the bath temperature above 43 °C serves as another biomarker of functional TRPV1 activity [14,17]. Following a rapid increase in the bath temperature from 21.5 °C to ≈43 °C at 240 s, the fluorescence ratio rose from 0.1042 ± 0.0008 at t = 200 s (n = 20) to 0.2226 ± 0.0121 and reached a peak at t = 300 s (n = 20, *** *p* < 0.001) (Figure 3a). Notably, these transient increases occurred during the initial 60 s after applying the heated bath solution. This phase was followed by a full recovery of the f340 nm/f380 nm ratio to 0.0992 ± 0.0016 at t = 600 s (n = 20, *** *p* < 0.001) (Figure 3a). In the next set of experiments, the cells were pre-incubated with 1 mmol/L of L-carnitine for 30 min. After heating above 43 °C, the f340 nm/f380 nm ratio was only 0.1175 ± 0.0036 at t = 300 s (n = 32, ^###^ *p* < 0.001), and a reduction was observed even below the baseline level to 0.0817 ± 0.0012 at t = 600 s (n = 32; ^###^ *p* < 0.001) (Figure 3b). With 3 mmol/L of L-carnitine, its inhibitory effect was like that induced by 1 mmol/L of L-carnitine (0.1110 ± 0.0011 at t = 300 s; n = 16, ^###^ *p* < 0.001) (Figure S3e). In summary, an increase in bath temperature induces a reversible response that reflects expression of a heat-sensitive TRPV1 variant in HCE-T cells. (Figure 3c and Figure S3f).

### 2.6. L-Carnitine Blocks Hypertonicity-Induced Corneal Epithelial Cell Shrinkage

**Figure 4:**
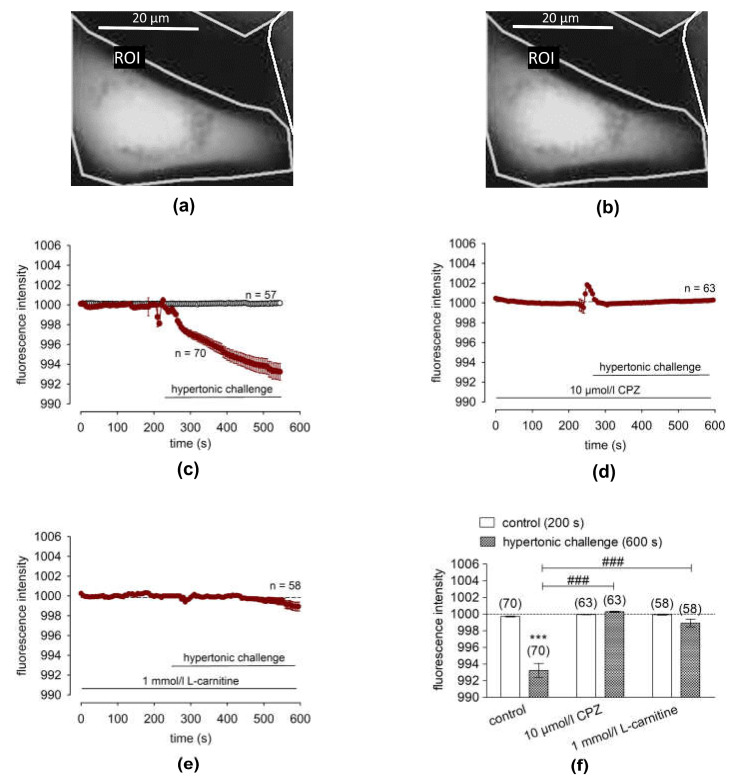
L-carnitine suppressed hypertonicity-induced cell shrinkage in HCE-T cells. (**a**) Fluorescence microscopic image (excitation wavelength 488 nm, emission wavelength 520 nm) of a calcein/AM-loaded HCE-T cell before and after a hypertonic challenge. Scale bar = 20 µm. The region of interest (ROI) is shown by a border. The image shows an HCE-T cell before hypertonicity (control) (t = 0 s). (**b**) The same HCE-T cell after the hypertonic challenge (450 mosmol/L) (t = 550 s). A minor reduction in cell size could be observed in the fluorescence microscope. (**c**) A hypertonic challenge (450 mosmol/L), indicated by the horizontal bar, led to a reduction in fluorescence intensity which was proportional to a reduction in the cell volume (n = 70, filled circles). A control measurement using an isotonic solution (316 mosmol/L) did not change the fluorescence intensity (n = 57; open circles). (**d**) Pretreatment with the TRPV1 antagonist CPZ (10 µmol/L) clearly attenuated this effect (n = 63, filled circles). (**e**) Pretreatment with L-carnitine (1 mmol/L) had a similar effect to CPZ (n = 58, filled circles). (**f**) Summary of the experiments with CPZ and with L-carnitine. Bars represent mean values ± SEMs of fluorescence intensity at 200 s (normalized baseline control values) and 600 s (the effect at the end of the observation period). The numbers of cells measured in both experiments are indicated in the brackets above the bars. The asterisks (*) designate significant reductions in cell volume following hypertonic challenge (t = 600 s; n = 70; *** *p* < 0.001; paired data. The hashtags (#) indicate statistically significant differences in fluorescence intensities between the hypertonic challenge (control, n = 70) and 10 µmol/L of CPZ (n = 63) or 1 mmol/L of L-carnitine (n = 58) (both t = 600 s; ^###^ *p* < 0.001; unpaired data), respectively.

To confirm that L-carnitine blocks TRPV1 activation by inhibiting hypertonicity-induced cell volume shrinkage, the effects of exposure to a 450 mosmol/L hyperosmotic stress on apparent cell volume in the presence and absence of 1 mmol/L of L-carnitine in calcein/AM-loaded cells were compared. Figure 4 shows a representative calcein-/AM-loaded cell before (A) and after the (B) hypertonic challenge.

Figure 4a shows representative recordings of the fluorescence intensity obtained from 58–70 cells in at least three independent measurements, which were recorded every 5 s for 600 s. Their levels were normalized to 1000, which was the average fluorescence intensity of most cells in this condition. Notably, the cells shrank rapidly at the beginning of the solution change, which may have been stress-induced or due to manual pipetting. A slight shrinkage occurred upon the application of the hypertonic challenge (≈450 mosmol/L) since the fluorescence intensity fell from 999.7 ± 0.05 to 993.2 ± 0.85 (arbitrary units) (Figure 4c, filled circles, n = 70), while a control measurement with an isotonic solution (316 mosmol/L) did not change the fluorescence intensity, indicating a constant cell volume (Figure 4c, open circles, n = 57). When TRPV1 was inhibited with 10 µmol/L of CPZ, the 450 mmol/L hypertonic challenge did not change the fluorescence intensity (Figure 4d, filled circles, n = 63, negative control), indicating that CPZ blocked cell shrinkage. Like CPZ, 1 mmol/L of L-carnitine prevented cell shrinkage (Figure 4e, n = 58). Taken together, pretreatment with either 1 mmol/L of L-carnitine or the TRPV1 channel blocker CPZ suppressed hypertonic challenge-induced cell volume shrinkage (Figure 4f, CPZ: ^###^ *p* > 0.001, L-carnitine: ^###^ *p* < 0.001). This correspondence between the effects of CPZ and L-carnitine validates that L-carnitine obviated shrinkage induced TRPV1 activation.

## 3. Discussion

### 3.1. Modes of Suppression of TRPV1 Activation by L-Carnitine

Functional TRPV1 variant expression was documented based on the expression of two different established biomarkers that accompany sensitivity to capsaicin. L-carnitine blocked CAP-induced increases in the Ca^2+^ influx, which agrees with previous reports in HCjEC and HCK [34,35]. The TRPV1 variant expressed in HCE-T cells is like its counterpart in HCK [35,37] but differs from the N-terminal variant of TRPV1 in that it is insensitive to CAP. Instead, the TRPV1 variant expressed in the non-excitable cell types HCE-T and HCjEC is sensitive to CAP, whereas the N-terminal TRPV1 variant is required for mediating osmosensory transduction in neurons [18]. The results of our study confirmed that L-carnitine is an effective osmoprotectant against hypertonicity-induced TRPV1 activation since preincubation with 1 or 3 mmol/L of L-carnitine blocked osmotic stress TRPV1 activation in HCE-T cells (Figure 1, Figure 2 and Figure 3, S1 and S2). Regarding CAP-induced TRPV1 activation, 3 mmol/L of L-carnitine was a less effective inhibitor than 1 mmol/L of L-carnitine (Figure S3a). One tenable explanation for the smaller decline with a slightly higher L-carnitine concentration may stem from a non-selective cytotoxicity effect that may cause the plasma membrane Ca^2+^ influx to increase. On the other hand, this lesser degree does not detract from the overall result of our study since in another experiment, the CAP-induced Ca^2+^ increase could be nearly fully blocked (Figure S1b).

**Figure 5:**
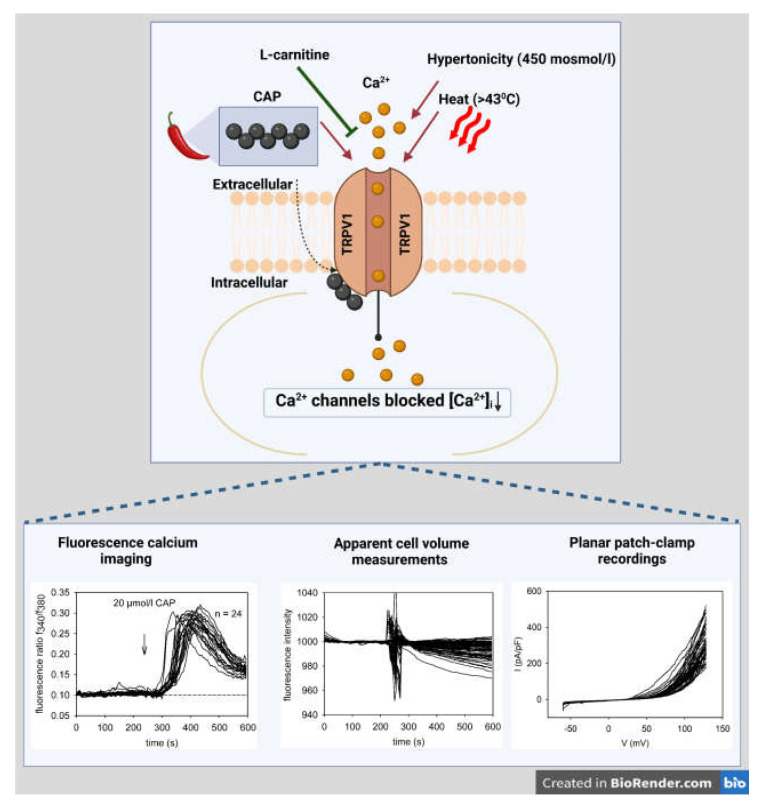
TRPV1 channel model accounting for how L-carnitine inhibits TRPV1 activation in HCE-T. Ca^2+^ channels such as TRPV1 can be selectively activated by CAP (Figure 1), hypertonicity (Figure 2) [41], or heat (>43 °C) (Figure 3) and blocked by L-carnitine (Figure 1, Figure 2 and Figure 3). Three different methods were used to validate the inhibitory effect of L-carnitine, such as single-cell fluorescence calcium imaging, cell volume measurements and planar patch-clamp recordings (lower panels) (created in Biorender.com, accessed on 21 July 2023).

L-carnitine (1 mmol/L) was already shown to suppress TRPV1 functional expression in excitable corneal nociceptive innervated neurons [38,39,40]. Our current study is unique since either 1 or 3 mmol/L of L-carnitine blocked each of the three different hallmarks of TRPV1 activation in HCE-T cells, as illustrated in Figure 5.

As TRPV1 activation contributes to DES symptomology, it is pertinent to identify agents such as L-carnitine that blocks hypertonic-induced TRPV1 activation (Figure 4). The similar inhibitory effects of 1 and 3 mmol/L of L-carnitine are likely attributable to their direct interactions with plasma membrane-delimited TRPV1 binding sites because the patch-clamp recordings show that they had similar inhibitory effects on the ionic currents. Therefore, the L-carnitine-mediated suppression of TRPV1 activity suggests that this effect stems from its selective inhibition of hypertonicity-induced cell shrinkage.

In patch-clamp recordings, an imposed large electrochemical gradient established by 2 mmol/L of Ca^2+^ in the external bath and a Ca^2+^-free internal medium promoted increases in the inward Ca^2+^ influx that were suppressed by L-carnitine (Figure S2c). Although an increase in Na^+^ influx may also contribute to increases in whole-cell currents due to the high Na^+^ concentration in the external measuring solution, there is a correspondence between the inhibitory effects of L-carnitine on the ionic currents and the calcium imaging data. This agreement suggests that L-carnitine is Ca^2+^-sensitive rather than Na^+^-sensitive, although a modulation of the Na^+^ influx by L-carnitine through TRPV1 cannot be fully excluded. On the other hand, Na^+^ is not a second messenger, and there are no other studies showing that L-carnitine mediated changes in Na^+^ influx. The L-carnitine inhibitory effect is instead attributable to the suppression of Ca^2+^ influx through an interaction with the TRPV1 pathway [34]. In contrast, to the absolute outward currents, the relative outward currents (% of control) were at partially lower levels compared to the relative inward currents (Figure S2d,e), suggesting the importance of the absolute inward currents borne by the Ca^2+^ influx. Furthermore, anion channel currents (Cl^−^) can be excluded for the most part since an isosmotic Na^+^-gluconate substitution did not change the outward currents in HCE-T [14].

### 3.2. L-Carnitine Suppresses Hypertonicity-Induced Cell Shrinkage and TRPV1 Activation

Like CPZ, L-carnitine suppressed hypertonicity-induced cell shrinkage by blocking TRPV1 activation [17,24]. L-carnitine was shown to have osmoprotective properties against hypertonic challenge-induced HCE-T cell shrinkage and apoptosis [42]. Figure 2 shows that the 450 mosmol/L hypertonicity-induced Ca^2+^ increase corresponds to those in HCK- [35], HCjEC- [34] and TRPV1-transfected HEK293 cells [17], as well as to increases leading to increases in inflammatory cytokine release in HCE-T cells [24]. CPZ blocked the hypertonicity induced Ca^2+^ increase (Figure 2b), confirming that TRPV1 activation underlies increases in the Ca^2+^ influx. L-carnitine (1 mmol/L) blocked the hypertonicity-induced cell shrinkage and, in turn, prevented an increase in intracellular Ca^2+^ levels (Figure 2c). A total of 3 mmol/L of L-carnitine (Figure S3c, d) also suppressed hypertonic-induced increases in the Ca^2+^ influx, which agrees with previous studies in HCK [35] and HCjEC [34]. Such an inhibition is indicative of a protective effect of L-carnitine against hypertonicity-induced Ca^2+^ overload, which is in line with another study that showed that L-carnitine suppresses the development of dry eye symptomology in a mouse model of dry eye [43]. As shown by Pan et al. (2011) [24], TRPV1 inhibition by CPZ also suppresses hypertonicity-induced increases in proinflammatory cytokine release in HCE-T cells [24]. Therefore, the L-carnitine-mediated inhibition of TRPV1 activity may be of therapeutic value in suppressing TRPV1-induced increases in proinflammatory cytokine release in DES. The similar inhibitory effects of L-carnitine on increases in TRPV1 activity induced by cell shrinkage, thermal activation or ligand binding confirm that L-carnitine is an effective inhibitor of hypertonicity-induced cell volume shrinkage, which, in turn, blocks TRPV1 activation.

### 3.3. L-Carnitine Suppresses Heat-Induced TRPV1 Activation

L-carnitine blocked TRPV1 activation induced by raising the bath temperature above 43 °C, which is another hallmark of the functional expression of TRPV1 [21,44]. Although this is a rather unphysiological condition and could influence other Ca^2+^-sensitive proteins following the heat shock-mediated apoptosis of corneal cells [45], HCE-T cells normally survive short heat pulses, even those above 50 °C, that elicit the heat-induced activation of TRPV2 [14]. Since the heat pulses were only transiently applied to the HCE-T cells, heat-induced toxicity was not a likely complication, as proven by full recovery after heat-induced Ca^2+^ transients occurred (Figure 3a). Exposure to either 1 or 3 mmol/L of L-carnitine substantially suppressed the heat-induced intracellular Ca^2+^ increase (Figure 3b,d and Figure S3e,f), as could already be shown in HCK [35]. However, the HCK cells were more compromised by the Ca^2+^ overload than HCE-T cells since in that study, the heat-induced Ca^2+^ increases were irreversible, and L-carnitine had a smaller inhibitory effect in HCK than in HCE-T.

### 3.4. L-Carnitine Suppresses Hypertonicity Induced Cell Volume Shrinkage

Hyperosmotic stress induced declines in calcein-derived fluorescence and mediated TRPV1 activation (Figure 4a–c). CPZ acted as an osmoprotectant by blocking osmotic-induced shrinkage and TRPV1 activation (Figure 4d). The blocking of TRPV1 activity by L-carnitine is likewise relevant since similar to CPZ, it could also suppress osmotic cell shrinkage (Figure 4e,f). Interestingly, an opposite effect was observed when TRPV1 expression was downregulated using TRPV1 siRNA in HCjEC. Furthermore, TRPV1 knockdown via TRPV1 siRNA may induce compensatory upregulations of other TRP isoforms (e.g., TRPM7 or TRPV4/AQP4 complex) that alter the regulation of Ca^2+^ homeostasis [46,47].

### 3.5. Clinical Impact

A deficiency in lachrymation or increased evaporation at the ocular surface can contribute to the development of DES pathology [48]. Clinically, tear film osmolarity is elevated in a significant proportion of DES patients, which is an important parameter that can also be used as a marker of its progression [49]. Pescosolido et al. reported that the L-carnitine concentrations in tear samples were substantially lower in DES patients than in healthy subjects. Based on this difference, they suggest that L-carnitine-containing lubricants may contribute to preventing the adverse effects of exposure to tear film hyperosmolarity in DES [50]. L-carnitine is used as an osmoprotectant adjuvant to treat DES in a clinical setting [7,27,28,51]. Baudouin et al. suggested that osmoprotectant usage in treating DES may directly protect cells against hyperosmolarity and interrupt the vicious cycle underlying DES pathophysiology [28,48]. Previous studies concur with the current study showing that L-carnitine suppresses hyperosmotic-induced cell volume shrinkage in HCE-T cells, retinal pigment epithelium, or conjunctival epithelium [34,52,53]. While L-carnitine was proven to reduce the levels of pro-inflammatory cytokines such as IL-6 and IL-8 [23], Khajavi et al. and Turan et al. attributed this effect to an interaction of L-carnitine with TRPV1 in HCjEC [34] and HCK [35]. On the other hand, DES is also accompanied by neurosensory abnormalities. Fakih et al. evaluated the effectiveness of TRPV1 blockade using CPZ to alleviate the ocular pain, neuroinflammation, and anxious behavior associated with severe DES [54]. Their study provides insight into the effectiveness of TRPV1 antagonist instillation in alleviating abnormal corneal neurosensory symptoms induced by severe DES. Notably, the Ca^2+^ response patterns resulting from inhibition of TRPV1-mediated hypertonicity-induced increases by CPZ were like those resulting from exposure to L-carnitine. In a multicenter, open-label observational study in Germany, it was shown that eye drops containing L-carnitine (Optive^®®^) were well tolerated and improved the DES symptoms, including reducing ocular pain in patients after 2 to 4 weeks [9]. Therefore, additional studies ought to be undertaken to evaluate the effectiveness of L-carnitine in reducing ocular pain.

## 4. Materials and Methods

### 4.1. Materials

Capsaicin and capsazepine were obtained from Cayman Chemical Company, Ann Arbor, MI, USA. Supplements for cell culture were ordered from Life Technologies Invitrogen, Karlsruhe, Germany or Biochrom GmbH, Berlin, Germany. Accutase was purchased from PAA Laboratories, Pasching, Austria. All the other reagents, including L-carnitine, were purchased from Sigma-Aldrich, St. Louis, MO, USA.

### 4.2. Cell Culture

A simian virus 40 (SV40)-immortalized human corneal epithelial cell line, HCE-T [55], was kindly provided by Friedrich Paulsen and Fabian Garreis (Institute of Anatomy, University of Nuremberg, Nuremberg, Germany). The HCE-T cells were cultured in Dulbecco’s modified Eagle medium DMEM/HAMs F12 at a ratio of 1:1, supplemented with 10% fetal calf serum (FCS) and antibiotics in a humidified 5% CO_2_ incubator at 37 °C [14,55]. The cells were passaged routinely using accutase to disperse the epithelial sheets when they reached subconfluence.

### 4.3. Fluorescence Calcium Imaging

HCE-T cells grown on coverslips were preincubated with 1 μmol/L of fura-2/AM (1 μL per ml, using a stock solution of 1 mmol/L) at 37 °C for 20–40 min. After the incubation period, the coverslips containing the cells were thoroughly rinsed with a Ringer-like (control) solution (RLS) containing (in mmol/L): 150 NaCl, 6 CsCl, 1 MgCl_2_, 1.5 CaCl_2_, 10 D-glucose and 10 HEPES (pH ≈ 7.4; osmolarity ≈ 316 mosmol/L). The coverslips were placed into a bath chamber and immediately rinsed with 1.5 mL of RLS under a fluorescence microscope (Olympus BX50WI; Olympus Europa Holding GmbH, Hamburg, Germany) equipped with a cellSens Dimension 2.3 software-controlled high-powered fluorescence LED light source (LED-Hub by Omikron, Rodgau–Dudenhoven, Germany), a high-resolution digital camera (Olympus XM10) and a peristaltic pump P-1 (Pharmacia, London, UK), which was connected to the bath chamber in a dark room at room temperature (RT) (~23 °C). In case of heat stimulation, a heated RLS at a temperature above 43 °C replaced the room temperature medium. These changes were monitored with a digital thermometer attached to the bath chamber (Voltcraft, Conrad Electronic SE, Hirschau, Germany). Single cells were selected and designated as regions of interest (ROIs) using the Olympus cellSens Dimension 2.3 software (Olympus Europa Holding GmbH, Hamburg, Germany), and they were excited via the use of alternating illumination at 340 and 380 nm in 5 s intervals through the Olympus cellSens Dimension 2.3 software-controlled interface (Olympus U-RTC). The fluorescence fura-2 emission was recorded at 510 nm (120 frames per experiment for a total experimental time of 10 min). The fluorescence ratio of the two wavelength traces, f340 nm/f380 nm, correlates directly with the changes in intracellular Ca^2+^ level ([Ca^2+^]_i_), as described by Grynkiewicz et al. [56]. The fluorescence ratios were calculated using Olympus cellSens Dimension 2.3 software and normalized (control set to 0.1), drift corrected (if applicable) and averaged (with error bars) using the TIDA version 5.22 for Windows (HEKA Electronik GmbH, Lambrecht/Pfalz, Germany). The results are depicted as mean traces of the f340 nm/f380 nm ratio ± SEM values, with n-values indicative of the number of measured cells per data point. The effects of L-carnitine were evaluated following a 30 min pre-incubation time. Amounts of 1 and 3 mmol/L of L-carnitine were used (Appendix A), which is similar to ranges used in numerous previous studies [10,34,35,57]. Drug stock solutions were prepared with DMSO and diluted in RLS so that the final DMSO concentration was below 0.1% in the working solution and had no detectable influence on Ca^2+^ regulation [14]. A hypertonic solution (≈450 mosmol/L) was applied, which was prepared by supplementing isotonic RLS with 130 mosmol/L D-mannitol.

### 4.4. Apparent Cell Volume Measurements

An Olympus BX50WI fluorescence microscope, in conjunction with an XM10 digital camera (both Olympus, Europa Holding GmbH, Hamburg, Germany), monitored the fluorescence emissions of the calcein-loaded HCE-T cells. The cells on coverslips were loaded with 1 µmol/L of calcein/AM (Cayman, Ann Arbor, MI, USA) in the growth medium at 37 °C in an incubator with 5% CO_2_ for 30–40 min. A loading of 1 µmol/L calcein-/AM was to monitor changes in fluorescence intensity that are directly related to the apparent cell volume [58]. This relationship was previously confirmed using a hypertonic challenge in different corneal cell types, including HCE-T cells [34,41]. The calcein fluorescence excitation wavelength at 494 nm and emission wavelength at 517 nm were measured using an ultra-bandpass filter set (500/534 nm bandpass). A LED-HUB with a 505 nm high-power LED with a peak wavelength at 495 nm was used as the light source (Omikron, Rodgau-Dudenhofen, Germany). After rinsing with an isotonic RLS (316 mosmol/L), the coverslips were again superfused in the same RLS in the bath chamber (control) for 4 min. After that, the aforementioned hypertonic medium was applied in the presence or absence of 1 mmol/L of L-carnitine.

### 4.5. Statistical Data Analyses

Paired data were probed for normality according to Kolmogorov–Smirnov, and Student’s *t*-test assessed the statistical significance of paired data if they passed normality. Alternatively, the Wilcoxon matched-pairs test was instead used if they failed normality. Likewise, statistical significance was determined for unpaired data using Student’s t-test if they passed normality or with the Mann–Whitney-U test if they failed normality testing. Probabilities of *p* < 0.05 (indicated by asterisks for paired data (*) and hash tags (#) for unpaired data) were considered significant. Statistical tests were performed, and diagrams were created using SigmaPlot, version 12.5, for Windows (Systat Software, Inc., Point Richmond, CA, USA) as well as GraphPad Prism software, version 5.00, for Windows (La Jolla, CA, USA). The number of replicates [n] is shown in each case in brackets, near the traces or bars. *p* values indicating different levels of significance, i.e. * or # for *p* < 0.05, ** or ## for *p* < 0.01, and *** or ### for *p* < 0.01. All values are given as means ± standard errors of the mean (SEM) (error bars in both directions).

## Data Availability

The data presented in this study are available on request from the corresponding author. The data are not publicly available due to privacy limitations.

## Supplementary Materials

The following supporting information can be downloaded at: https://www.mdpi.com/article/10.3390/ijms241411815/s1.

## Appendix A

### Methods Concerning Supplementary Materials

#### Photomultiplier Fluorescence Calcium Recording

Time-dependent changes in the free intracellular calcium concentration ([Ca^2+^]_i_) were recorded with the fluorescent dye fura-2/AM. The HCE-T cells were pre-incubated with culture medium containing 2 µmol/L of fura-2/AM for 15–45 min at 37 °C. Prior to the experiments, the cells were rinsed with a Ringer-like (control) solution containing 150 mmol/L of NaCl, 6 mmol/L of CsCl, 1 mmol/L of MgCl_2_, 10 mmol/L of glucose, 10 mmol/L of HEPES and 1.5 mmol/L of CaCl_2_ at pH 7.4 (300 mosmol/L) to remove any dead cells or cellular debris. The fura-2 fluorescence in the washed cells was measured at room temperature using a digital imaging system (TILL Photonics, Munich, Germany) in connection with a detector (Hamamatsu Photonics, Sunayama-cho, Naka-ku, Hamamatsu City, Shizuoka Pref., Japan) and a fluorescence microscope (BW50I, Olympus, Hamburg, Germany). The Fura-2 fluorescence response signals were alternately excited at 340 and 380 nm wavelengths, and emissions were detected from cell clusters every 500 ms at 510 nm. The fluorescence ratio (f_340nm_/f_380nm_) was calculated as a relative index of the changes in [Ca^2+^]_i_. The results are shown as mean traces of the fluorescence ratio f_340nm_/f_380nm_ ± SEM values, with n-values indicating the number of experiments per data point.

#### Planar Patch-Clamp Recordings

Electrophysiological recordings were obtained using a high-throughput semi-automated planar patch clamp setup in whole-cell mode (Port-a-Patch; Nanion^®®^, Munich, Germany). Measurements were controlled and recorded with PatchMaster software (Version 2.73 from HEKA, Lambrecht/Pfalz, Germany). Single-cell suspensions were prepared in an external measuring solution containing (in mmol/L) 140 NaCl, 4 KCl, 1 MgCl_2_, 2 CaCl_2_, 5 D-Glucose monohydrate and 10 HEPES-acid at pH 7.4 and 298 mosmol/L. The single-cell suspension droplet was placed on top of a small aperture on a microchip. Single cells were driven into the aperture via negative pressure, which was supplied by a Patch-Control 4.1.18 software-controlled pump (Nanion^®®^, Munich, Germany). Inside the chip, 5 µL of a calcium-free internal measuring solution, containing (in mmol/L) 50 CsCl, 10 NaCl, 60 CsF, 20 EGTA, and 10 HEPES (pH ≈ 7.2 and ≈288 mosmol/L), were applied. The extracellular solution was applied onto the chip with a small pipette. The membrane capacitance (15.34 ± 2.86 pF; n = 12) and access resistance (8.42 ± 1.2 MΩ; n = 12) were calculated using PatchMaster 2.8 software (HEKA Elektronik GmbH, Lambrecht/Pfalz, Germany). Series resistances, fast and slow capacitance transients as and leak currents were compensated for by the patch clamp amplifier (HEKA Elektronik, Lambrecht/Pfalz, Germany). All experiments were performed at room temperature, ≈23 °C, unless stated otherwise. The holding potential (HP) was set to 0 mV to eliminate any possible contribution of voltage-dependent Ca^2+^- channel activity. Every 5 s, a voltage ramp protocol (voltage change from −60 to +130 mV) for 500 ms was applied. Current recordings were leak-subtracted, and cells with leak currents above 100 pA were excluded from data analysis. The currents were normalized with respect to the cell membrane capacitance to obtain current density plots (pA/pF).
