# Supplementary material for: L-Carnitine Suppresses Transient Receptor Potential Vanilloid Type 1 Activation in Human Corneal Epithelial Cells
Source: Int J Mol Sci. 2023 Jul 23;24(14):11815. doi: 10.3390/ijms241411815 (PMC10380586; doi:10.3390/ijms241411815)
Supplement: Supplementary file 1 [file ijms-24-11815-s001.zip › ijms-2509539-supplementary.pdf]

Supplementary Materials

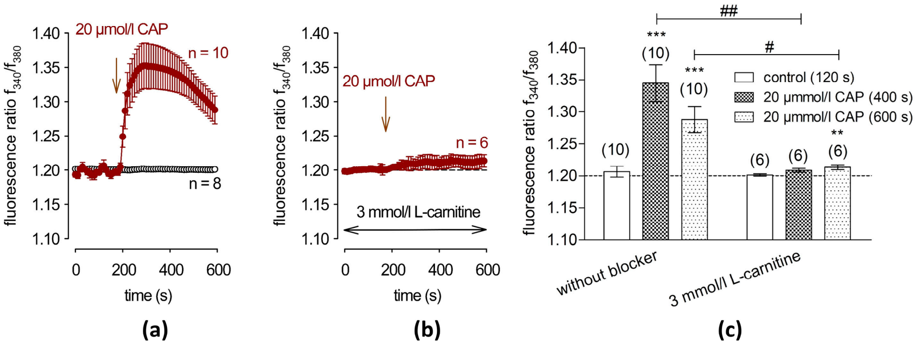

Fig. S1: L-carnitine leveled calcium influx due to TRPV1 activation by capsaicin in HCE-T cells. (a) Exposure to capsaicin (20 µmol/l) at the time point indicated by the arrow led to a strong calcium influx (n = 10 coverslips, filled circles) whereas a control measurement showed a constant Ca^{2+} baseline (n = 8 coverslips, open circles). (b) Pretreatment with L-carnitine (3 mmol/l) abolished this effect (n = 6 coverslips). (c) Statistical evaluation of the experiments shown in (a) and (b). The asterisks (*) indicate statistically significant differences with capsaicin (n = 10; $p < 0.05$ at the minimum; paired tested). The hash marks (#) indicate statistically significant differences of fluorescence ratios with and without L-carnitine ($p < 0.05$ at the minimum; unpaired tested).

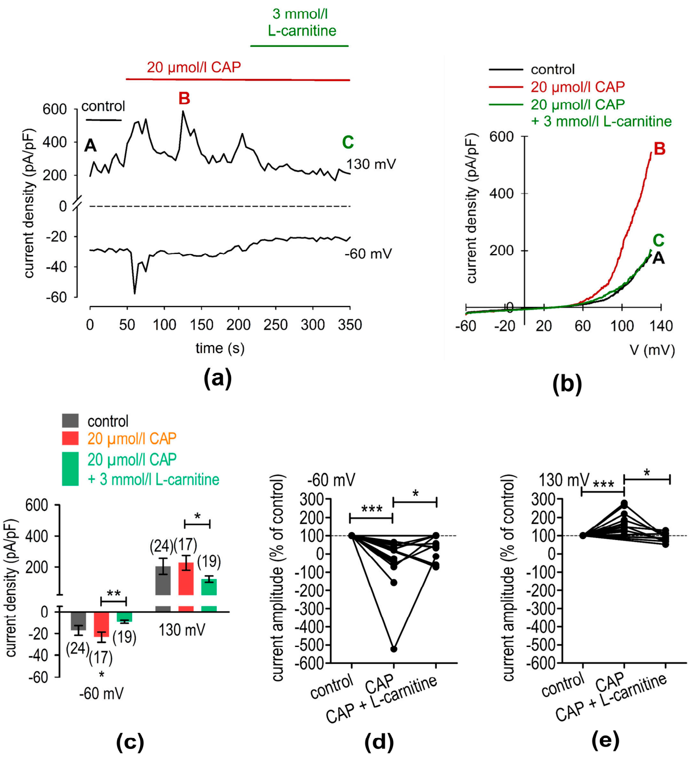

Fig. S2: L-carnitine inhibits CAP-induced whole-cell currents in HCE-T cells. (a) Time course recording of whole-cell currents at -60 mV (lower trace) and 130 mV (upper trace). The currents were normalized to cell membrane capacitance (current density; pA/pF). 20 µmol/l CAP led to current increases, whereas application of 3 mmol/l L-carnitine suppressed these rises. (b) Original traces of current responses to voltage ramps shown before application (black curve, labeled as A), as well as during application of 20 µmol/l CAP (red curve, labeled as B), and after adding 3 mmol/l L-carnitine (green curve, labeled as C). (C) Statistical analysis of the patch-clamp recordings of CAP-induced current increases with and without L-carnitine. The asterisks (*) designate statistically significant differences of CAP-induced increases of in- and outward whole-cell currents without and with L-carnitine (n = 12 - 24, *$p < 0.05$ at the minimum; unpaired tested). (d) Maximal inward current amplitudes induced by a voltage step from 0 mV to -60 mV are shown in percent of control values before application of 20

µmol/l CAP (control set to 100 %). The CAP-induced inward currents increased (n = 16, ***$p < 0.001$; one sample t-test) and could be suppressed by 3 mmol/l L-carnitine (n = 11, *$p < 0.05$; Wilcoxon matched pair test). (e) Same analysis as shown in (d) but concerning the outward currents at a voltage step from 0 mV to 130 mV. The CAP-induced outward currents increased (n = 16, ***$p < 0.001$; one sample t-test) and could be suppressed by 3 mmol/l L-carnitine (n = 11, *$p < 0.05$; paired tested).

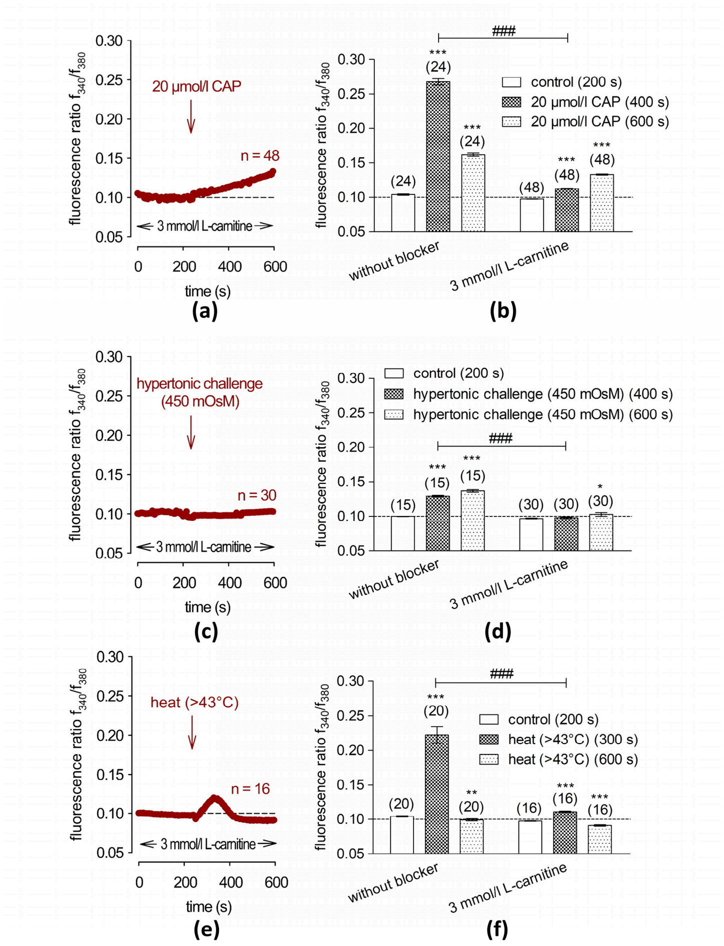

Fig. S3: L-carnitine (3 mmol/l) suppress CAP, hypertonicity, and heat-induced Ca^{2+} rises. The time dependent changes are shown as relative intracellular Ca^{2+} levels in fura2-loaded HCE-T cells. Data are represented as mean ± SEMs, n indicates the number of cells examined in this set of experiments. Dashed line represents the reference line for baseline value (0.1). Arrows indicate the extracellular application of CAP, hypertonicity, or heat at 240 s. (a) Representative graph of the TRPV1 blocking effect of 3 mmol/l L-carnitine on CAP induced Ca^{2+} influx (n = 48) (b) Statistical analysis of this effect. Bars represent mean values ± SEM of fluorescence ratio at 200 (control), 400 and 600 s (CAP). The numbers of cells measured in both experiments are indicated in brackets above the bars. The asterisks (*) indicate statistically significant differences with and without CAP (n = 24 to n = 48; ***$p < 0.001$; paired tested). The hashtags (#) refer to unpaired data with and without L-carnitine ($^{###}p < 0.001$). (c) 3 mmol/l L-carnitine completely blocked the hypertonicity-induced Ca^{2+} increase (n = 30). (d) Statistical analysis as shown in panel (b) but with hypertonic challenge instead of CAP. The asterisks (*) indicate statistically significant differences with and without hypertonic challenge (n = 15 to n = 30; *$p < 0.05$ at the minimum; paired tested). (e) 3 mmol/l L-carnitine completely blocked the heat-induced Ca^{2+} increase (n = 16). (f) The statistical analysis is similar as shown in panel (b) but with heat instead of CAP. The asterisks (*) indicate statistically significant differences with and without hypertonic challenge (n = 16 to n = 20; **$p < 0.01$ at the minimum; paired tested).
